# Fatal mobbing and attack of the common cuckoo by its warbler hosts

**DOI:** 10.1002/ece3.9649

**Published:** 2022-12-21

**Authors:** Huahua Zhao, Haixia Luo, Hanlin Yan, Gangbin He, Longwu Wang, Wei Liang

**Affiliations:** ^1^ School of Life Sciences Guizhou Normal University Guiyang China; ^2^ Ministry of Education Key Laboratory for Ecology of Tropical Islands, Key Laboratory of Tropical Animal and Plant Ecology of Hainan Province, College of Life Sciences Hainan Normal University Haikou China

**Keywords:** brood parasitism, common cuckoo, fatal attack, host mobbing, nest defense, oriental reed warbler

## Abstract

Nest defense is an effective strategy of hosts against parasites. Typically, hosts will aggressively attack brood parasites that approach or visit their nests, which can prevent the parasites from laying eggs or may even lead to the death of the parasites. Few previous studies have specifically reported such fatal cases involving brood parasites and have attributed the cause of death to either drowning or hypothermia after falling into the water following an attack from hosts. In this study, we recorded the process of multiple host individuals of the Oriental reed warbler (*Acrocephalus orientalis*) mobbing and attacking a female common cuckoo (*Cuculus canorus*) in the field. We discovered that the immediate cause of the cuckoo's death was the fatal physical damage resulting from the aggressive defense from the hosts, suggesting that frantic pecking and scratching by the hosts is the most proximate cause of mortality among egg‐laying female cuckoos. This finding enhances our essential understanding of the effectiveness of host attacks.

## Graphical abstract

We discovered that the immediate cause of the cuckoo's death was the fatal physical damage resulting from the aggressive defense from the Oriental reed warbler, suggesting that frantic pecking and scratching by the hosts is the most proximate cause of mortality among egg‐laying female cuckoos. This finding enhances our essential understanding of the effectiveness of host attacks.

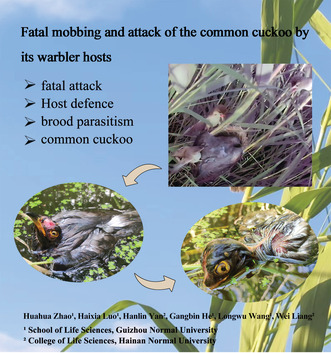

## 1 INTRODUCTION

The interaction between brood parasites and hosts in the avian brood parasitism system is one of the best model systems for studying coevolution (Davies, 2000, 2011; Soler, 2014). The common cuckoo (*Cuculus canorus*) (hereafter cuckoo) is currently the most widely and intensively studied brood parasitic species. Cuckoos always lay their eggs in the nests of other host birds and rely entirely on the hosts to incubate cuckoo egg and rear the nestling (Davies, 2000; Soler, 2017). The host will resist through strategies such as nest defense and egg rejection to reduce the high cost of parasitism (Feeney et al., 2014; Gloag et al., 2014; Peer, 2006; Trnka & Grim, 2014). Previous studies described that the process of cuckoo parasitism was secretive (Davies, 2000; Davies & Brooke, 1988). However, based on numerous video recordings in the field, it is summarized that the process of cuckoo parasitism is conducted openly as the hosts defend nest around all the times. The cuckoos will continue to forcefully lay eggs despite the hosts initiating strong attacks when they approach or visit the hosts' nests (Jelínek et al., 2021; Moksnes et al., 2000; Nakamura et al., 2005; Trnka & Prokop, 2012).

Nest defense is the frontline of host defense against parasitism (Feeney et al., 2012, 2014; Welbergen & Davies, 2009). When a parasite invades, a host usually attempt to prevent being parasitized through strategies such as calling, flapping of wings, scratching, tearing feathers, and pecking at the head or eyes to repel the parasite (Edwards et al., 1949; Jelínek et al., 2021; Wang et al., 2020). These attacks can cause the parasite to suffer severe injuries and even death (Gloag et al., 2013; Šulc et al., 2020). To date, seven cases of parasites dying near the hosts' nests have been reported. However, owing to the lack of video recording evidence in the field, the death of those parasites can only be presumed to be caused by host mobbing, and ultimately the exact cause of death cannot be determined (Loflin, 1982; Molnár, 1944). With the development and application of video recording, while studying the relationship between the great reed warbler (*Acrocephalus arundinaceus*) (GRW) and cuckoo, Šulc et al. (2020) incidentally recorded the process of a host attacking one cuckoo, which was later found dead on the surface of the water 20 m away from the nest. Based on their analysis and given that no specific injury was visible on the exterior surface of the cuckoo, the cuckoo was speculated to have fallen into the water after being attacked by the host and eventually died from drowning and hypothermia.

We conducted a field study related to the breeding of Oriental reed warblers (*Acrocephalus orientalis*) (ORWs) and cuckoos from June to August 2022 at Sifangtuozi Farm (46°12′ N, 123°84′ E), Bai Cheng City, Ji Lin Province, northeastern China. Local ORWs prefer to nest in reedbeds along riverbanks or ditches, and ORW was highly aggressive host to cuckoos (Chen et al., 2022; Wang et al., 2022; Yu et al., 2019), which even strongly attack the sparrowhawk (*Accipiter nisus*) in dummy experiments (Ma, Yang, & Liang, 2018). Cuckoos are the main parasites of ORWs and have a high parasitism rate of 34.3%–65.5% (Li et al., 2015; Wang et al., 2020, 2021; Yang et al., 2014, 2016). To record a video of cuckoo parasitism, we systematically searched for ORW nests throughout the study area and then mounted a microcamera (Uniscom‐T71, 70 × 26 × 12 mm; Mymahdi Technology Co., Ltd., Shenzhen, China) above the nest. We managed to record an instance of ORWs violently attacking a female cuckoo. Compared to the previous studies, our study recorded the entire process of the cuckoo being mobbed and killed by multiple hosts. This not only confirms that host attack is the immediate cause of the cuckoo's death but also enhances our essential understanding of host nest defense.

## 2 RESULTS

**FIGURE 1:**
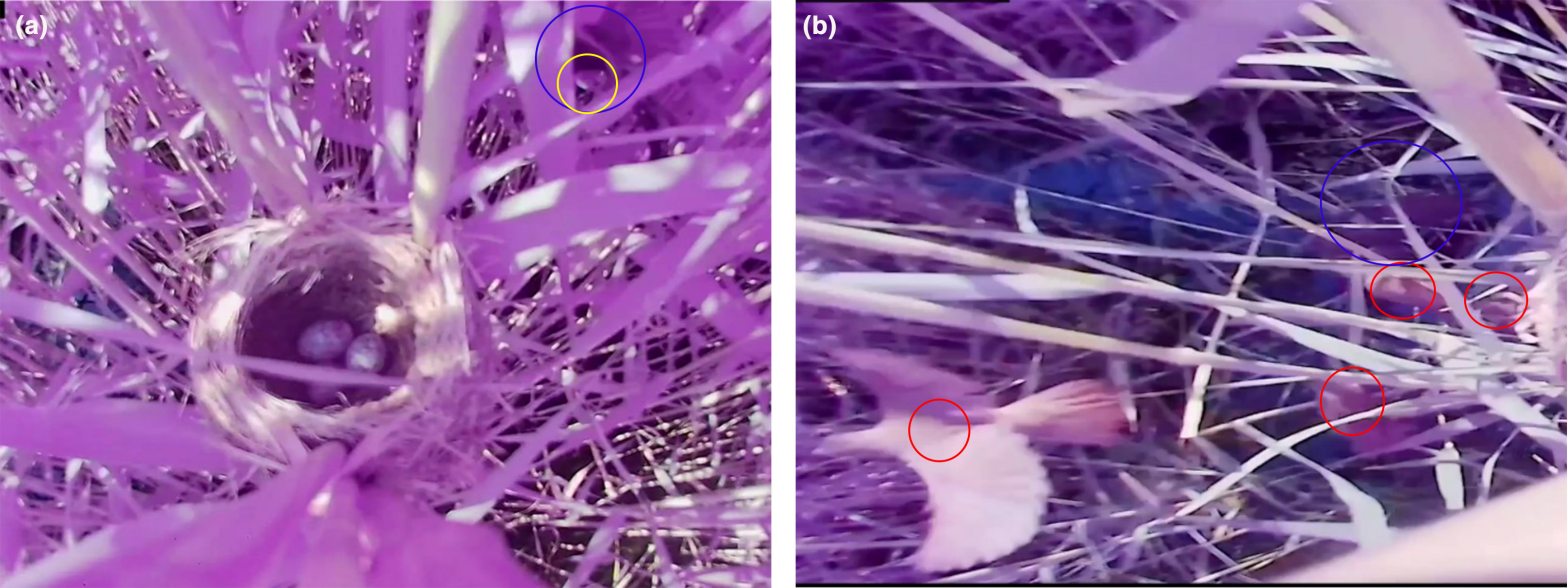
Fierce attack by the hosts (a refers to the common cuckoo laying its egg outside the host's nest under strong attack; the yellow circle marks the egg that is being laid, and the blue circle marks the common cuckoo that is under intense attack. b refers to multiple hosts are mobbing the common cuckoo; the red circles indicate oriental reed warblers, and the blue circle indicates the common cuckoo).

By analyzing the video recording, a female cuckoo was observed being aggressively attacked by the ORWs and falling into the water below the nest during a parasitic attempt at 15:37 on June 29, 2022. Meanwhile, the cuckoo was forced to lay its eggs outside the nest owing to the aggressive attack by the ORWs (Figure 1a; Video S1). Since the beginning of the attack, the hosts emitted loud and noisy alarm calls. We noticed that four to six hosts (Figure 1b) participated in the attack, which occurred in the form of mobbing. Some hosts jumped on the cuckoo's back; frantically tore feathers and pecked at the head or eyes with their bills; and flapped with their wings open while others swooped quickly toward the cuckoo from above one reed to perform a sneak attack and then quickly flew to another reed (Video S1).

**FIGURE 2:**
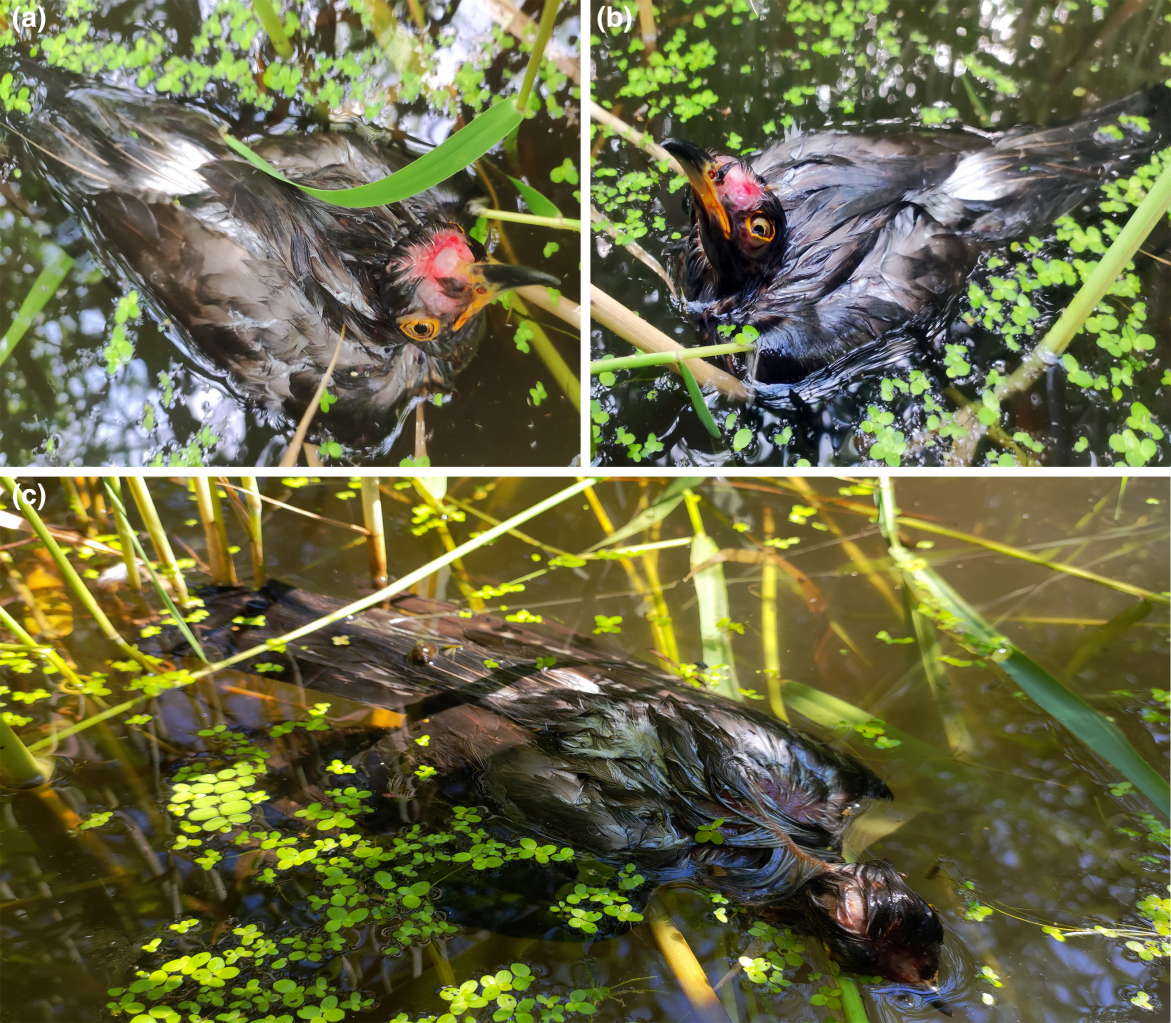
Images of the fatal wounds and death of the common cuckoo (a refers to the severe tearing and pecking of the head feathers and head epidermis of the cuckoo, leading to exposure of the skull; b refers to the common cuckoo with right eye severely pecked; and c refers to the cuckoo died in the water beneath the host's nest on June 30).

In addition, the host mobbing became more intense when the cuckoo tried to escape by flapping its wings in the water, causing the cuckoo to emit wailing cries (detail in Video S1, at 1 min 1 s). The attack lasted approximately 150 min and resulted in fatal injuries including the cuckoo's head feathers and scalp being completely ripped off, skull being exposed (Figure 2a), and left eye lacerated (Figure 2b). In the morning of June 30, 2022, the cuckoo was found dead in the water underneath the host nest (Figure 2c).

## 3 DISCUSSION

The video recording showed that the cuckoo emitted wailing cries and suffered fatal physical trauma from the frantic pecking and scratching by multiple ORWs, and eventually the prolonged and sustained aggressive mobbing led to the death of the cuckoo. However, drowning and dense reeds cannot be ruled out as alternative causes of the cuckoo's death.

Detection and attack by hosts are surprisingly common when cuckoos perform parasitism in these aggressive hosts (Jelínek et al., 2021; Moksnes et al., 2000; Wang et al., 2020). Previous studies have suggested that cuckoos choose to parasitize in the afternoon to avoid detection and attack by hosts while reducing the risk of egg rejection (Davies, 2000; Davies & Brooke, 1988). In addition, rapid egg laying is thought to be an evolutionary adaptation to a parasitic life (Soler, 2014, 2017). Furthermore, besides laying in the afternoon and laying rapidly, a growing number of experimental studies show that female cuckoos rely on bubbling calls to protect themselves from mobbing hosts (e.g., Marton et al., 2021; York & Davies, 2017). Generally, when a host is present, cuckoo parasitism will invoke attacks from the host which may cause injury to or even death of the cuckoo (Jelínek et al., 2021; Šulc et al., 2020; Wang et al., 2020). Currently, no studies have clearly stated the form of attack that could lead to the extreme outcome of the death of cuckoos (Šulc et al., 2020). In Japan, one cuckoo made a total of nine parasitic attempts, each of which was blocked by more than three GRWs. It failed in the first eight attempts and only barely succeeded in laying eggs at the ninth attempt (Nakamura et al., 2005). In other avian brood parasitism systems, a smooth‐billed ani (*Crotophaga ani*) was killed by the attack from seven hosts and died underneath the host nest (Loflin, 1982). In addition, three little friarbirds (*Philemon citreogularis*) and one black butcherbird (*Cracticus quoyi*) conducted a lethal attack toward a female Eastern koel (*Eudynamys orientalis*) (Jackson & Kyne, 2010). Apparently, a large number of hosts involved in attacking the parasites not only lowers the chances of successful parasitism but also may increase the likelihood of cuckoo death. Based on our study on the lethal process resulting from the mobbing of a cuckoo by four to six ORWs, we speculate that the effectiveness of attacking the parasites may be reflected in the dominance of host number.

The alarm call of a host usually can attract and provoke nearby conspecifics to participate in attacking a cuckoo (Welbergen & Davies, 2008), and the strength of nest defense is positively correlated with the number of attracted hosts (Grim, 2008). An isolated ORW's nest has been noticed to be more susceptible to parasitism (Ma, Yang, Liu, et al., 2018) as this hinders the attraction of a large number of hosts for a common attack. This suggests that the presence of a nearby host breeding cluster enhances the host nest defense. We also observed that none of the other host nests near our studied host nest was parasitized by cuckoos for a period after the study because of the death of the female cuckoo who probably is the owner of the area, although such an example has not been reported previously. In conclusion, our result is the first documentation that host mobbing can be a primary cause of death in cuckoos, and that a common defense is effective in deterring cuckoo parasitism.

## AUTHOR CONTRIBUTIONS

**Huahua Zhao:** Data curation (equal); methodology (equal); writing – original draft; writing – review and editing. **Haixia Luo:** Data curation (equal); methodology (equal). **Hanlin Yan:** Data curation; investigation. **Gangbin He:** Data curation; investigation. **Longwu Wang:** Investigation; supervision; writing – original draft; writing – review and editing. **Wei Liang:** Supervision; writing – review and editing.

## FUNDING INFORMATION

This work was supported by the National Natural Science Foundation of China (Nos. 31960105, 32260253 to L.W., 31970427, 32270526 to W.L.). L.W. was funded by the Guizhou Natural Science Foundation (No. ZK [2022]‐316), and W.L. was supported by the specific research fund of The Innovation Platform for Academicians of Hainan Province.

## CONFLICT OF INTEREST

The authors declare that they have no competing interests.

### OPEN RESEARCH BADGES

This article has earned Open Data and Open Materials badges. Data and materials are available at https://datadryad.org/stash/share/ApMtTH1NsiZYd5byxzMlzzd0l1Ek‐Nexp_nPiG3ANk4.

## Supporting information


Video S1.

## Data Availability

Supplementary material "Video S1. Process of Oriental reed warblers attacking the female common cuckoo" alongside this manuscript at the Dryad Digital Repository: https://datadryad.org/stash/share/ApMtTH1NsiZYd5byxzMlzzd0l1Ek‐Nexp_nPiG3ANk4.
