# Supplementary material for: Fatal mobbing and attack of the common cuckoo by its warbler hosts
Source: Ecol Evol. 2022 Dec 21;12(12):e9649. doi: 10.1002/ece3.9649 (PMC9772492; doi:10.1002/ece3.9649)
Supplement: Supplementary file 1 — Video S1. [file ECE3-12-e9649-s001.docx]

**We upload this file, in order to remove the video in original submission portal, which was 105 M, reached the allowed file size limit for submissions, so we need remove it in our author portal and then proceed further.**
